# Plasmonic Biosensors with Nanostructure for Healthcare Monitoring and Diseases Diagnosis

**DOI:** 10.3390/s23010445

**Published:** 2022-12-31

**Authors:** Tongge An, Jiahong Wen, Zhichao Dong, Yongjun Zhang, Jian Zhang, Faxiang Qin, Yaxin Wang, Xiaoyu Zhao

**Affiliations:** 1 College of Materials and Environmental Engineering, Hangzhou Dianzi University, Hangzhou 310018, China; 2 The College of Electronics and Information, Hangzhou Dianzi University, Hangzhou 310018, China; 3 Shangyu Institute of Science and Engineering, Hangzhou Dianzi University, Shaoxing 312000, China; 4 Institute for Composites Science Innovation, School of Materials Science and Engineering, Zhejiang University, Hangzhou 310027, China; 5 Zhejiang Laboratory, Hangzhou 311100, China

**Keywords:** plasmonic biosensors, nanoparticles, lithography methods, SERS, healthcare monitoring, disease diagnosis

## Abstract

Nanophotonics has been widely utilized in enhanced molecularspectroscopy or mediated chemical reaction, which has major applications in the field of enhancing sensing and enables opportunities in developing healthcare monitoring. This review presents an updated overview of the recent exciting advances of plasmonic biosensors in the healthcare area. Manufacturing, enhancements and applications of plasmonic biosensors are discussed, with particular focus on nanolisted main preparation methods of various nanostructures, such as chemical synthesis, lithography, nanosphere lithography, nanoimprint lithography, etc., and describing their respective advances and challenges from practical applications of plasmon biosensors. Based on these sensing structures, different types of plasmonic biosensors are summarized regarding detecting cancer biomarkers, body fluid, temperature, gas and COVID-19. Last, the existing challenges and prospects of plasmonic biosensors combined with machine learning, mega data analysis and prediction are surveyed.

## 1. Introduction

With continuous development of physics, materials, medicine, communication, computer and other disciplines, we have higher requirements and expectations for daily healthcare and disease diagnosis [1]. At present, the common method for daily detection of various physiological indicators (such as the concentration of glucose in the body) that can reflect human health is still blood examination [2]. This detection method that will create a wound not only brings pain to patients but also increases the risk of bacterial infection. For diagnosis of diseases, expensive detection methods are usually used, such as CT, B-ultrasound, pathological detection and so on, which can only play a role in the later stage of many diseases. Thus, more convenient, faster and affordable detection methods are imperative for improving the ability of disease prevention and early diagnosis [3].

In this context, plasmonic biosensors have been particularly favored in the fields of healthcare monitoring and disease diagnosis owing to their preponderance of low detection limit, low cost and convenience [4,5,6] The plasmonic biosensor is mainly based on surface plasmon polaritons (SPPs) and localized surface plasmon resonances (LSPRs), which are two plasmonic response modes excited by lasers of corresponding wavelength in noble metal (such as Au and Ag) nanostructures [7,8,9]. By interacting effectively with external radiation, conduction band electrons around nanostructures exhibit collective oscillation, which concentrates electromagnetic radiation into the space of nanostructures, confines light below the diffraction limit and achieves obvious local electromagnetic field enhancement [10,11]. In particular, the enhancement effect of the desired local electromagnetic field can be obtained by adjusting the size, shape, spacing and periodicity of nanostructures [12]. Thereby, the sensing sensitivity of biosensors is enhanced by boosting plasmon-mediated chemical reactions (PMCRs) and analyzing plasmon-enhanced molecular spectroscopy (PEMS) signals, including plasmon-enhanced Raman spectroscopy (PERS), plasmon-enhanced infrared spectroscopy (PEIRS) and plasmon-enhanced fluorescence (PEF) spectroscopy [13,14]. In addition, it can show more flexible and real-time sensing performance when the plasmonic structure is integrated on the optical fiber tip than on the plane substrate. Optical fiber with plasmonic sensing structure at the top can detect a small amount of analytes in the liquid environment and can even be inserted into the tissue to identify and locate malignant tumors in vivo [15].

In this review, we will briefly summarize the common manufacturing methods for fabrication of a plasmonic biosensing structure, such as chemical synthesis, direct writing lithography, nanosphere lithography and so on. Thereafter, we will present advanced applications of plasmonic biosensors in the fields of detection of cancer markers, gas, temperature, body fluid, COVID-19, etc., containing the principles, fabrications and prospects. To conclude, we will probe the future prospects of plasmonic biosensors combined with machine learning.

## 2. Fabrication of Sensing Structure in Plasmonic Biosensors

Sensing material with nanopatterned arrays is the most vital part of plasmonic sensors, which accomplishes the task of enhancing biosensor through its unique electrical or optical properties [16]. Thanks to rapid development of micro-nano manufacture and characterization technology, there are many possible fabricating methods for us to construct various sophisticated nanostructures, such as synthesis of nanoparticles, various lithography methods, deposition and etching. However, each manufacturing method has its advantages and limitations so that we need to decide which one or several to choose and combine with each other in order to construct complex nanostructures.

### 2.1. Synthesis of Nanoparticles

Noble metal nanoparticles (NPs) are widely used in plasmonic structure because of its relatively simple process. Usually, by using metal–salt solution (e.g., HAuCl_4_ for Au and AgNO_3_ for Ag) and appropriate reductant (e.g., citrate sodium), it is possible that noble metal NPs are reduced directly from metal–salt solution [17,18]. Noble metal NPs within a certain size range can be obtained by controlling the ratio of reductant to noble metal. However, NPs prepared in this way are easy to agglomerate, and it is challenging to precisely adjust the volume and distribution of the particles. Therefore, researchers have explored more manufacturing methods of noble metal NPs to adjust the shape, volume and distribution of NPs more accurately on this basis [19,20,21].

**Figure 1:**
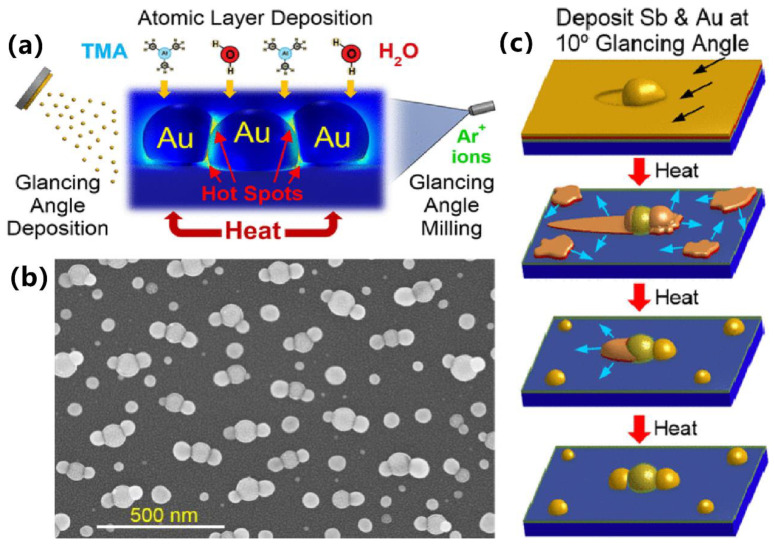
Plasmonic trimer structure based on nanoparticles and its synthesis process. (**a**) The abridged general view of the aligned trimers composed of Au nanostructures. (**b**) The SEM images of the assembled trimers. (**c**) The schematic diagram of manufacturing process. Reproduced with permission from reference [24]. Copyright 2022 American Chemical Society.

Solid-state dewetting is a technology to obtain noble metal NPs on a large area by using vacuum deposition and annealing [22]. The size and distribution of NPs can be adjusted by tuning the deposition thickness, annealing time and annealing temperature [23]. Depositing a sacrificial layer of Sb before Au can significantly increase the characteristic length scale of Au NPs in a recent report; hence, the volume and distribution of Au NPs can also be manipulated by adjusting the thicknesses of Au and Sb, thus tuning the plasmonic properties to an extent. One example is the aligned trimers composed of Au nanostructures proposed by Zachary’s group (Figure 1a,b), which was achieved via sequential deposition of Sb and Au, high-temperature assembly process, PVD, ALD, GLAD and wet-etching processes (Figure 1c). They also extended the approach to manufacturing periodic trimer and dimer arrays through nanoimprint lithography technology [24].

### 2.2. Various Lithography Methods

To fabricate more complicated nano-scale structure arrays, hard and soft lithography approaches, which form a desired two-dimensional pattern then convert the pattern into metallic plasmonic arrays, have been widely adopted, including EUV lithography, direct writing lithography, nanosphere lithography, nanoimprinting, DNA-assisted lithography, etc. [25].

#### 2.2.1. EUV Lithography

EUV lithography is the most widely used and mature technology in micro and nano fabrication industry, and it utilizes a photomask to form a two-dimensional exposed pattern on substrate coated with photoresist [26]. The stepwise procedure begins with spinning and coating a thin and uniform photoresist on the surface treated wafer. This is followed by baking the photoresist in advance, which improves adhesion between photoresist and substrate. The wafer is then positioned and exposed by lithography machine. Subsequently, it is placed in the developer to remove unwanted photoresist. The so-formed pattern is transferred to the wafer after baking, etching and photoresist removal [27]. Mask lithography can be processed in large quantities, but its working resolution is limited by optical diffraction. The smaller plasmonic structure arrays we want, the more precise lithography machine is required, so expensive equipment raises the threshold of lithography technology.

#### 2.2.2. Direct Writing Lithography

In addition to photomask lithography, there are also many direct writing lithography methods that write patterns directly on the substrate without mask, including electron-beam lithography (EBL) [28,29], focused ion-beam (FIB) [30] and direct laser writing (DLW) [31,32,33].

The desired pattern can be written by EBL, FIB or DLW on the substrate or photoresist directly. EBL and DLW write patterns on photoresist and then transfer the patterns to the substrate by plasma-reactive ion etching (RIE). Due to the large ion mass, FIB can directly write patterns on the sample surface without photoresist. However, since direct writing lithography is a kind of processing method based on point scanning, although it has high flexibility and resolution for graphic design, it also leads to limitations, such as slow producing speed and small producing area. Among three methods, EBL has the highest resolution, FIB has the least processing steps and DLW has the highest throughput. Additionally, the high cost of direct writing lithography is an impediment for industrial production. To address these problems, several groups have employed holographic lithography, nanosphere lithography or nanoimprint lithography to construct plasmonic structures over large areas.

**Figure 2:**
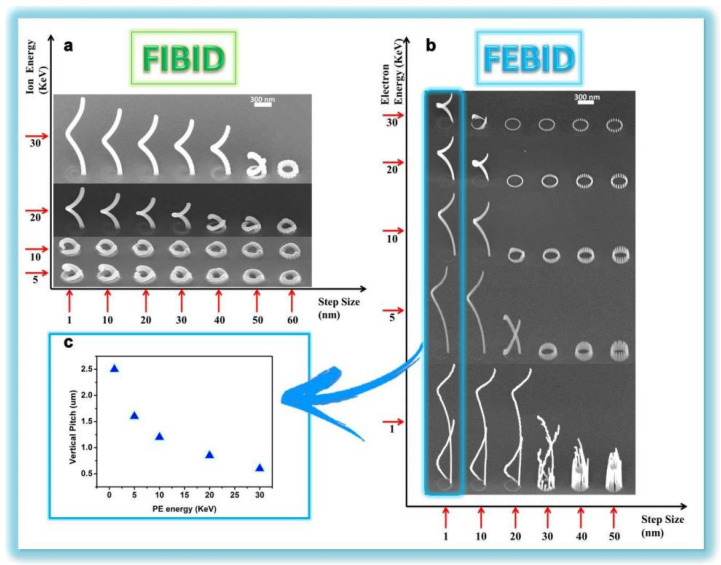

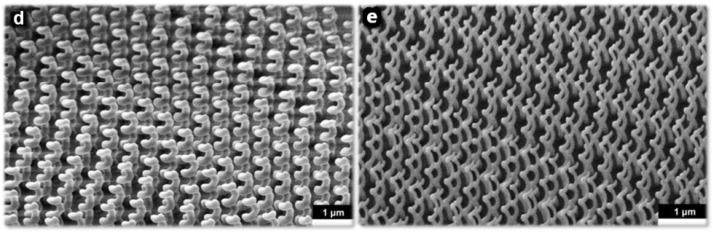
Plasmonic spiral structure and its fabricating process based on Focused Ion−beam (FIB). (**a**) SEM representation of the 3D FIBID nanohelix growth as a function of ion energy and step size. (**b**) SEM representation of the 3D FEBID nanohelix growth as a function of ion energy and step size. (**c**) In FEBID process, the single loop nanohelix VP strongly depends on the primary electron acceleration voltage. (**d**) Array of 20 × 20 3D chiral plasmonic nanohelices fabricated by FIBID. (**e**) Array of 40 × 40 3D chiral plasmonic nanohelices realized by FEBID. Reproduced with permission from reference [34]. Copyright 2015 American Chemical Society.

Recently, Esposito et al. prepared a three-dimensional chiral plasmonic spiral structure (Figure 2) by focused ion-beam-induced deposition (FIBID) and focused-electron-beam-induced deposition (FEBID) [34]. The complex three-dimensional spiral nanostructure shows strong chiral plasmonic activity. The chiral structure fabricated by FIBID indicates broadband polarization selection is about 600 nm and maximum dissymmetry factor up to 40% in the near infrared region. The chiral structure fabricated by FEBID bespeaks a highly selective dichroic band shifted towards shorter wavelengths and a maximum dissymmetry factor up to 26% in the visible range. Moreover, a finite difference time domain (FDTD) model is established that can design plasmonic structures to achieve desired optical response by controlling the actual size and geometric characteristics of the surface plasmonic spiral metamaterial [35].

#### 2.2.3. Holographic Lithography

The desired pattern is formed by interference of multiple laser beams in holographic lithography, so holographic lithography is also called interference lithography [36]. Adjusting the quantity and efficiency of the interference laser, incident angle, frequency and exposure time, one-dimensional or two-dimensional structures with different shapes, periods and heights can be produced [37]. The dense plasmonic structures can be fabricated on a large scale without being out of focus by reason of the photoresist covered on the substrate being exposed to interference area simultaneously [38]. Holographic lithography can only be used to prepare arrayed structures; therefore, it is suitable for periodic, rapid and low-cost mass production of plasmonic biosensors.

**Figure 3:**
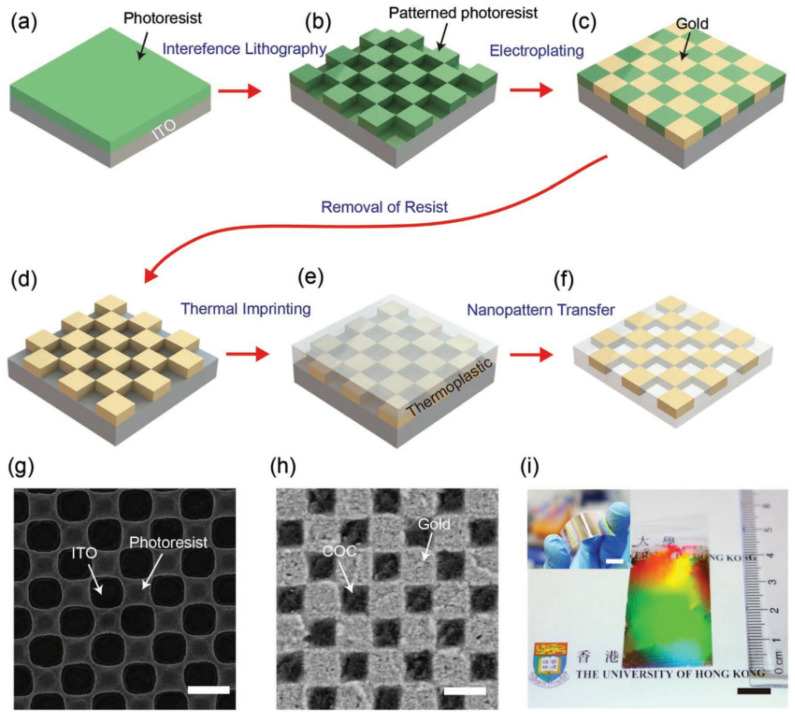
Plasmonic checkerboard structure and its fabricating process based on Holographic Lithography (HL). (**a**–**f**) The schematic diagram of manufacturing gold nanocheckerboard structure (ITO: indium tin oxide). (**g**) SEM image of the nanocheckerboard pattern prepared by HL on photoresist. (**h**) SEM image of the nanocheckerboard structure inserted in a cyclic olefin copolymer (COC) film. (**i**) Photographs of the plasmonic film with gold nanocheckerboard metasurfaces. Reproduced with permission from reference [39]. Copyright 2019 WILEY-VCH Verlag GmbH & Co. KGaA, Weinheim.

A gold nano-checkerboard structure based on holographic lithography for biomolecular sensing is developed by Cai’s group. The specific preparation method is shown in Figure 3. First, a layer of photoresist is spun on the indium tin oxide (ITO) substrate, and then a nano-checkerboard pattern is formed on the photoresist by holographic lithography technology. The next step is depositing a layer of Au. After removing the photoresist, the gold nano-checkerboard is heated and pressed into the thermoplastic film. The structure shows a high sensitivity of 435.1 nm RIU^−1^ in plasmonic refractive sensing applications at 570–610 nm wavelength and achieves the function of biosensors by monitoring the LSPR peak shift of the polymer interaction between bovine serum albumin (BSA) and anti-BSA protein [39].

#### 2.2.4. Nanosphere Lithography (NSL)

NSL is a widely used manufacturing method to produce large-scale biosensor array structures with a relatively simple process [40]. Ordered and uniform plasmonic array structures can be prepared based on closely arranged micro-spheres formed by capillary force between colloidal spheres [41,42]. Colloidal spheres can be self-assembled into monolayer or multilayer and then combined with deposition, RIE, wet etching, annealing and other methods to produce a variety of complex periodic two-dimensional or three-dimensional plasmonic structures [43,44,45], such as nano holes, nano bowls, nano columns, nano cones and so on [46].

Compared to other photolithography methods, NSL can manufacture a large area of plasmonic sensing structures, which has lower production cost and is more practical [47,48]. In particular, the shape of nano-structures made by NSL is variable, which can be fabricated as three-dimensional nano-structures to a certain extent; thus, it increases the flexibility of structure design [49,50]. Yet, although NSL does not require photomask, which simplifies the manufacturing steps, there are possibly local defects in plasmonic arrays due to the fact that the colloidal spheres are not fully aligned in local areas in the self-assembly process, and it will affect the uniformity of the plasmon sensing structures.

**Figure 4:**
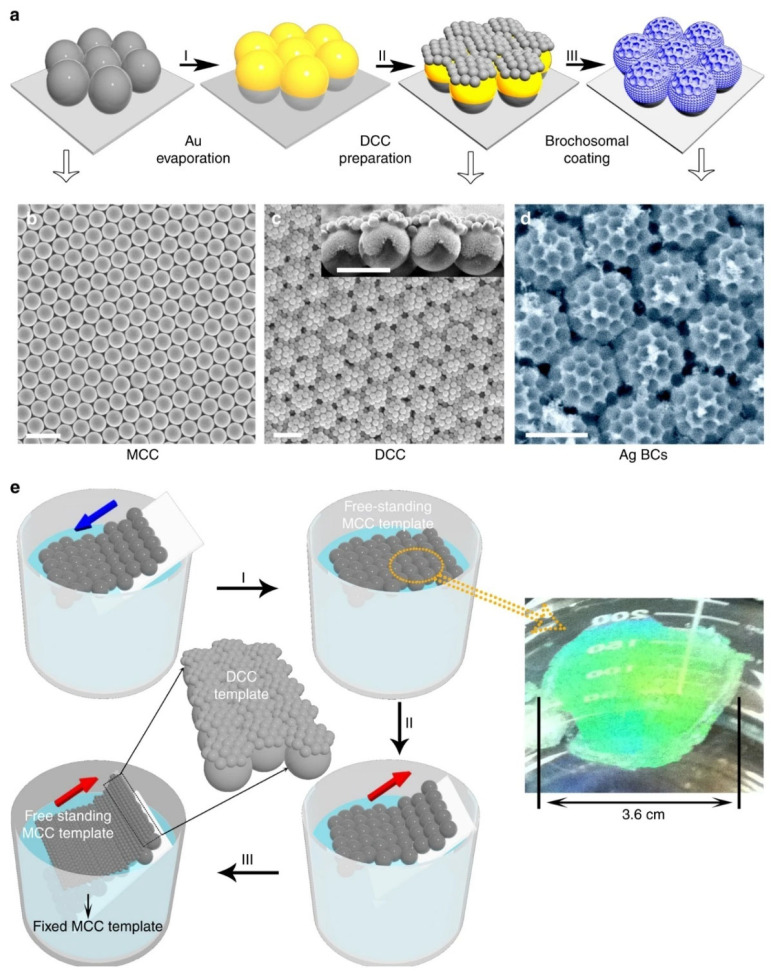
Plasmonic football-like structure and its fabricating process based on Nanosphere Lithography (NSL). (**a**) The preparation process of the football-like microstructure prepared by NSL. (**b**) SEM images of the monolayer mold. (**c**) SEM images of the double-layered mold. (**d**) SEM images of the football-like microstructure. (**e**) The schematic diagram of mold fabrication. Reproduced with permission from reference [51]. Copyright 2017 Springer Nature.

Recently, Yang and co-workers demonstrated a football-like antireflective surface microstructure inspired by leafhoppers. This unique structure is obtained by electrochemical deposition on the template of double-layer colloidal ball. The specific preparation process is shown in Figure 4. The first layer of template is obtained by self-assembly of a single layer of larger microspheres on the interface between water and air. After depositing a layer of gold film, a layer of smaller microspheres is self-assembled on the first layer of template to obtain a double-layer colloidal sphere template. Then, silver is deposited to fill the gap of the template and connect with the gold film. Finally, the microspheres are removed to obtain a unique soccer-like plasmon structure. It has been proved that the structure prepared by the above method for 2 μm thick silver can reflect <~1% any wavelength at 250–2000 nm wavelength. The excellent anti-reflective function can be applied to plasmonic sensing devices [51].

In order to realize integration of plasmonic nanostructure and optical fiber, Manago’s group fabricated three highly ordered plasmon arrays: close-packed array (CPA), sparse array (SA) and ball removal (SR) by nanospheres lithography technology and researched their effects on three representative biological probes: ultra-low-molecular-weight molecule biphenyl-4 mercaptan (BPT), Bovine serum albumin (BSA) and red blood cells [52]. In addition, they optimized the Raman readout system of the optrode to achieve efficient illumination and signal acquisition. In contrast, the effect of direct measurement on a flat substrate in a wide spectral range (900–1800 cm^−1^) is weakened.

#### 2.2.5. Nanoimprint Lithography (NIL)

NIL makes templates by EBL or other lithography methods and then transfers the structures on the templates to the photoresist on the substrate by mechanical pressing [53,54]. After the photoresist is cured, the plasmonic structures can be prepared by combining with deposition, etching and other methods [55,56]. NIL has benefited many applications in plasmonic biosensors owing to its advantages of low cost, high throughput and high resolution. Among them, the reusability of the templates reduces the production cost, and the sensor structures can be obtained fast in a large scale [57,58]. Nevertheless, the three advantages of NIL are highly dependent on the templates manufactured by EBL lithography, so it is suitable for mass production of simple three-dimensional structures. In addition, due to the mechanical extrusion processing that NIL utilized, there will always exist some residues of photoresist to prevent damage on the substrate from the mold, but the residues will affect the morphology of the structures ultimately prepared.

**Figure 5:**
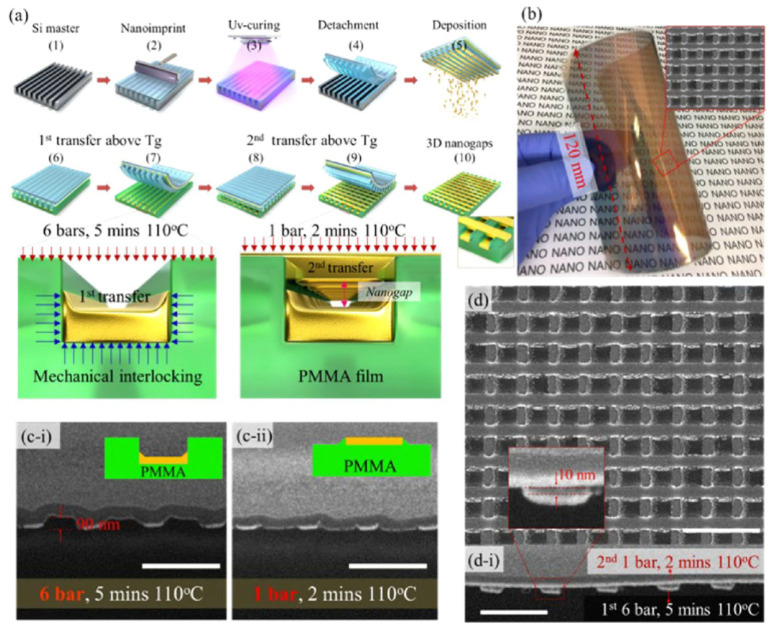
Plasmonic multilaminar nanopores structure and its fabricating process based on Nanoimprint Lithography (NIL). (**a**) The manufacturing process of multilaminar nanopores prepared by NIL. (**b**) The photo of the film with multilaminar nanopores. (**c-i**) and (**c-ii**) The sectional view of multilaminar nanopores manufactured under different conditions. (**d**) and (**d-i**) SEM overlooking and cross-sectional images of multilaminar nanopores. Reproduced with permission from reference [59]. Copyright 2021 American Chemical Society.

Zhao and his colleagues proposed a method to prepare multilaminar nanopores using layer by layer nanoimprinting, which simply achieved controlling the gap of nanopores on a large area (Figure 5). They used nanoimprinting and UV curing to create polymer templates and then bonded the template deposited with metal with the flexible substrate. The template and substrate are heated at 110 °C to transfer the metal to the substrate. The template is rotated to the direction perpendicular to the previous direction, and the metal transfer steps are repeated layer by layer to obtain multi-layer nanopore structure. The size of the nanogaps can be easily controlled by adjusting the pressure, heating time and heating temperature [59].

**Figure 6:**
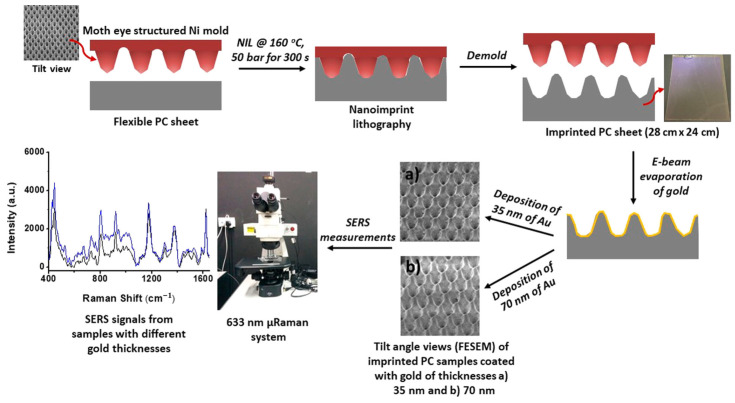
The schematic diagram of manufacturing Au nanocones as SERS substrate. Reproduced with permission from reference [60]. Copyright 2018 American Chemical Society.

In addition, a flexible plasmonic sensor composed of Au nanocones based on nanoimprinting technology is demonstrated by Suresh’s group. The geometry of the Au nanocones is adjusted by the mold of NIL, and the gaps between nanocones are controlled by the thickness of the deposited gold (Figure 6). The plasmonic structure obtained by this fabricating method has high sensitivity for surface-enhanced Raman spectroscopy (SERS) to detect analytes, and its advantages of high resolution, high throughput and low cost make available for mass production in industry [60].

#### 2.2.6. DNA-Assisted Lithography (DALI)

DALI method is to assemble different patterns and structures by DNA origami and then transfer the structures to the substrate by combining deposition, RIE, wet etching and other steps [61]. DNA origami is programmable so that the desired three-dimensional structures with high-precision can be designed flexibly, which is a capability that is unattainable when using the conventional nano-manufacturing techniques. However, it is demanding to control the distribution of structures precisely in the self-assembly process, so it is necessary to combine traditional lithography technologies to form periodic array structures. Further, the fabricating steps of DALI are too sophisticated to be easily applied for experiments and productions.

**Figure 7:**
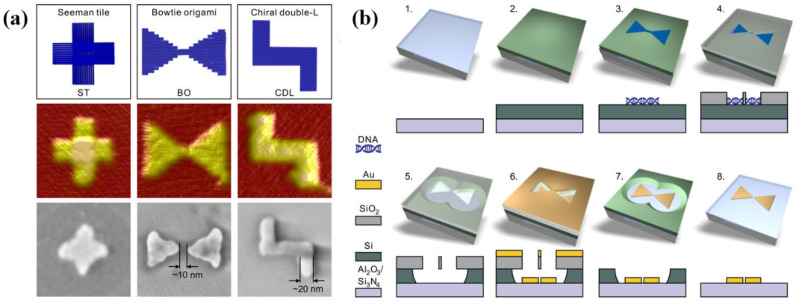
DNA origami designs and its fabricating process based on DNA-Assisted Lithography (DALI). (**a**) Designed three plasmonic structures prepared by DALI and their AFM image and SEM image. (**b**) The procedure of manufacturing bowtie origami. Reproduced with permission from reference [62]. Copyright 2018 American Association for the Advancement of Science.

Combining the high resolution and multifarious structure of DNA origami with conventional lithography techniques, Shen’s group reported a DALI method that can create elaborate structures on the scale of 10 nm. The specific preparation process is as follows (Figure 7): (1) an amorphous silicon layer is deposited on top of the transparent sapphire (Al_2_O_3_)/silicon nitride (Si_3_N_4_) substrate by PECVD. (2) The DNA origami nanostructures are drop-casted on the substrate treated by oxygen plasma. (3) The silicon dioxide (SiO_2_) layer is selectively grown on the bare silicon that is out of DNA structure by CVD. (4) Silicon is etched by RIE with mask that SiO_2_ is regarded as. (5) Depositing a layer of metal to obtain metal DNA structures. (6) Removing SiO_2_ and residual Si. Authors showed the practicability of this method by manufacturing metal nanostructures with a chiral plasmonic response and bowtie-shaped nanoantennas [62].

After pattern templates are prepared by various photolithography methods, the patterns on the templates need to be transferred to the substrate, which requires RIE, deposition, wet etching and other methods for auxiliary manufacturing. Most photolithography methods require their assistance to prepare metal plasmonic structures. Due to most lithography technologies only being able to produce two-dimensional patterns on photoresist, patterns must be transferred to the substrate and manufactured to the third direction, so RIE is required to transfer patterns and fabricate patterns in the depth direction during the manufacturing process. RIE is isotropic etching, so it can retain the pattern better and transfer it to the substrate. In addition, for plasmonic structures, the position of the gap as “hot spot” is vital; therefore, RIE is very critical as a step controlling the size of gap manufacturing in NSL. Generally, metal is deposited on the substrate by magnetron sputtering, thermal evaporation or electrochemistry. In most structures, the metal is deposited perpendicular to the substrate surface to obtain the same or complementary shape as template. However, changing the direction of substrate placement can make the metal grow obliquely, which may result in some unique plasmon structures. Wet etching is usually used to remove the sacrificial layer of Si or SiO_2_. Yet, it should be noted that there is a characteristic of anisotropy in the alkaline solution wet etching of monocrystalline silicon. Thus, wet etching can be used to fabricate three-dimensional structures, such as grooves and pits, on the silicon substrate.

We expect a cheap and fast manufacturing method that can control the size, shape and distribution of the structures in a large area, yet advantages and limitations exist at the same time in each fabricating method. Hence, it is critical to select the appropriate manufacturing techniques for every specific application. Moreover, a combination of multiple manufacturing methods may be more effective than a single technique for sophisticated structures.

## 3. Applications of Plasmonic Biosensors

In clinical health monitoring and disease diagnosis, it is of importance to detect patients’ physiological signals and biomarkers [2]. More and more biosensors have been developed in order to keep abreast of patients’ health status and screen diseases more conveniently. The various plasmonic nanostructures described in the previous section have been widely exploited in diverse plasmonic biosensors. Some typical application scenarios of plasmonic biosensors will be shown in this section, including detection of cancer markers, gas, temperature, COVID-19 and so on.

### 3.1. Detection of Cancer Markers

Cancer has a high mortality rate, high metastasis rate and high recurrence rate and remains among the most common causes of death worldwide [63]. The traditional methods for diagnosing cancers include pathological tissue biopsy, magnetic resonance imaging (MRI), computed tomography (CT), B-ultrasound, chest X-ray, endoscopy and so on. The effect of these tests on early detection of cancers is very limited, and some detection methods are not only expensive but also painful to patients. Therefore, it is necessary to carry out rapid and effective detection in the early stage of cancer, which can not only achieve the purpose of early detection and treatment but also improve the patients’ medical experience. Cancer markers exist in blood, cells or body fluids, which reflect the existence and growth of cancer cells [64,65]. They can be timely diagnosed before symptoms occur and greatly improve the survival rate of cancer patients [66,67,68].

**Figure 8:**
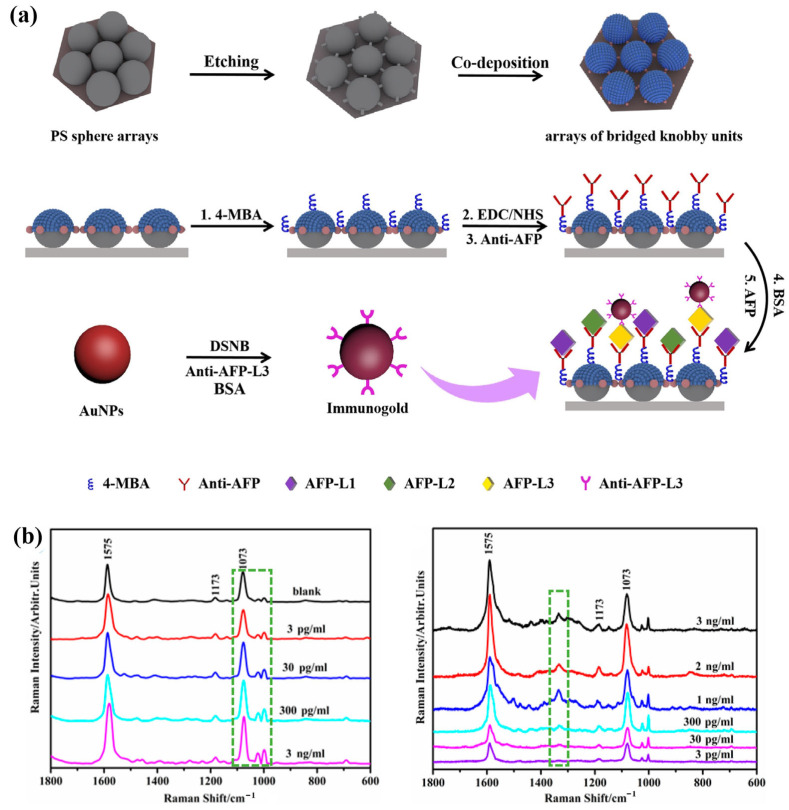
Fabricating process and SERS spectra of plasmonic structure for tumor marker detection. (**a**) The fabrication process of the bridged knobby units. (**b**) The SERS spectrum of α-fetoprotein (AFP) and α-fetoprotein-L3 (AFP-L3) in different concentrations in the biosensor. Reproduced with permission from reference [70]. Copyright 2020 Elsevier B.V.

Surface-enhanced Raman spectroscopy (SERS) can provide unique molecular “fingerprint” information by identifying the shift and intensity change in spectral signal [69]. The preponderances of high sensitivity and simple operation make it have broad application prospects in detection of cancer markers. For example, in the recent report of Cheng et al., a kind of array of bridged knobby units is fabricated by combining NSL, RIE and magnetron sputtering for detection of hepatocellular carcinoma (HCC) (Figure 8). This unique structure significantly enhances the sensitivity of detection owing to changing the distribution of hot spots between plasmonic structures and quantitatively analyses the concentrations of α-Fetoprotein (AFP) and AFP-L3 according to the change in frequency and strength in SERS. Using these plasmonic biosensors for detection of HCC is faster, more convenient and sensitive. The detection limit of AFP and AFP–L3 is reduced to 3 pg/mL in this work [70].

Some researchers have shown that, since the exosomes from tumor cells can carry the information of tumor cells, exosomes show potential for early detection and diagnosis of tumors. Recently, Lee and his colleagues developed a Au nanopillar SERS biosensor with ultra-high sensitivity and high specificity for quantitative determination of exosomal miRNAs from breast cancer cells. Highly uniform plasmonic Au nanopillars generate dense hot spots; thus, coupling of local plasma fields is enhanced and targeting miRNAs hybridized is specifically locked with nucleic acid (LNA) probes. The sensor can distinguish single base mismatch in miRNA molecules and shows an exceedingly low detection limit as little as 1 am, 100 times lower than other SERS methods to detect miRNA, a wide dynamic range (1 am to 100 nm), multiplex sensing capability and sound miRNA recovery in serum. The detection level of exosomal miRNAs from breast cancer cells by this sensor is consistent with the detection level of traditional QRT-PCR. More importantly, this SERS sensor can classify the molecular subtypes of breast cancer cell lines in light of the expression mode of exosomal miRNAs, which indicates that it provides a new method for classification of molecular subtypes of breast cancer [71].

Further, Hyungsoon and co-workers described a label-free high-throughput approach based on surface plasmon resonance (SPR) for quantitative analysis of exosomes. Detection of exosomes (nPLEX) by this plasmonic biosensor is based on light transmission of periodic nanopore arrays instead of total internal reflection commonly used in industry since the detection depth (<200 nm) of periodic nanopores arrays and the size of exosome (nPLEX) match each other. In order to biosensor can be more convenient to operate, they combined the sensing device with the integrated circuit to design a sensor chip, which can display the spectral shift or intensity change in direct proportion to the target labeled protein level by targeting specific exosome binding. Compared with traditional methods, it can continuously and real-time monitor molecular binding with highly sensitivity and without label [72].

### 3.2. Gas Biosensors

Toxic and dangerous gases in the environment and human health go hand in hand owing to not only their irritation of the upper respiratory tract but also the possibility of causing accidents. Therefore, detection of toxic gases in the living environment is very necessary for prevention of diseases and maintenance of human health [73]. Plasmonic sensors are also widely used in gas sensing, such as detecting hydrogen [74,75,76,77], nitrogen dioxide [78] and organic volatile gases [79,80].

Kyeorei and his colleague reported transparent nanopatterned chemiresistors composed of aligned 1D Au–SnO_2_ nanofibers, indium tin oxide (ITO) transparent electrodes and glass substrate for detecting toxic NO_2_ gas at room temperature under visible light illumination. The plasmonic NO_2_ sensor has exceedingly low coverage of sensing materials (≈0.3%), resulting in high transparency (≈93%) of the entire biosensor. In particular, thanks to the surface plasmon effect, the Au nanofiber arrays in the gas sensor significantly enhance the response signal without external heaters or light sources, and its detection limit is as low as 6 ppb [78].

**Figure 9:**
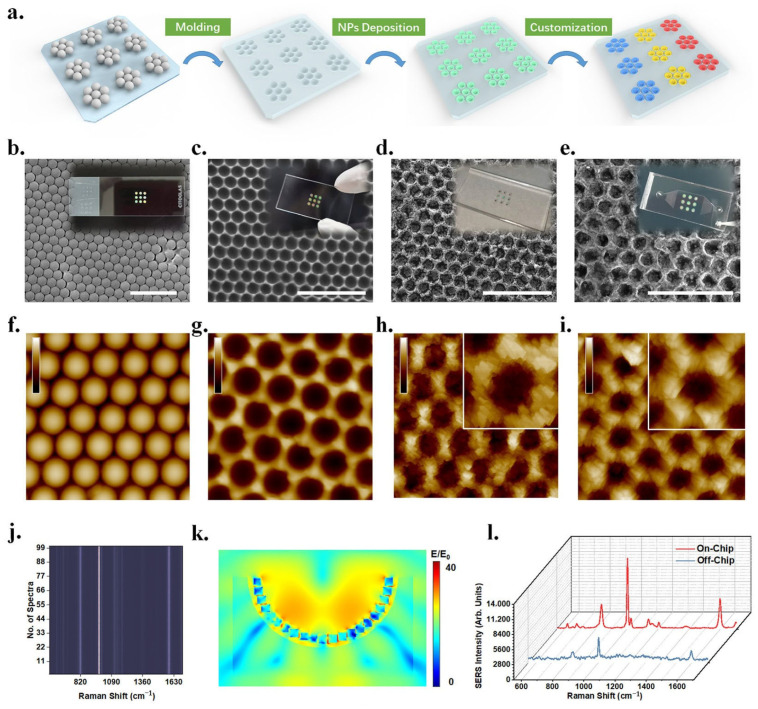
Construction and characterization of the plasmonic gas sensor with three different detection units. (**a**) Schematic diagram of fabricating the gas sensor. (**b**) SEM image of PS mold. (**c**) SEM image of PDMS substrate with nanopores. (**d**) SEM image of PDMS-substrate-deposited nanocubes. (**e**) SEM image of the substrate after deposition of Ti_3_C_2_T_x_ MXene. (**f**–**i**) AFM images corresponding to (**b**–**e**) diagram. (**j**) The stack image of SERS spectra of benzaldehyde at random regions. (**k**) Numerical simulation of the electromagnetic field in a single dent deposited bimetallic nanocubes. (**l**) SERS spectra of benzaldehyde obtained on the sensor and off the sensor. Reproduced with permission from reference [79]. Copyright 2022 American Chemical Society.

In order to achieve ultra-high sensitivity and high selectivity detection of multiple gases, combining with microfluidic chips and surface-enhanced Raman spectroscopy (SERS), Yang and his co-workers designed a plasmonic gas biosensor containing three different detection units to identify the gas through physical adsorption and chemical adsorption at the same time. The preparation step of plasmonic structure is to fabricate mold by NSL method first and then deposit bimetallic (Au@Ag@Au) nanocubes by self-assembly method (Figure 9). A gas biosensor with small volume, fast reading speed and low cost can simultaneously identify nine different gases with high sensitivity, high selectivity and high robustness. Moreover, the authors also claim that their sensors can maintain high selectivity without being disturbed by complex detection environments in indoor pollution detection and exhaled gas detection [79].

### 3.3. Temperature Sensor

Temperature sensing is vital in daily healthcare monitoring, disease diagnosis and complex medical solutions due to abnormality of human body temperature possibly being clinically confirmed as a major symptom of major public health events, such as SARS and COVID-19. Plasmonic temperature sensors can provide stable and sensitive temperature sensing signals, which has great development potential [81,82,83].

**Figure 10:**
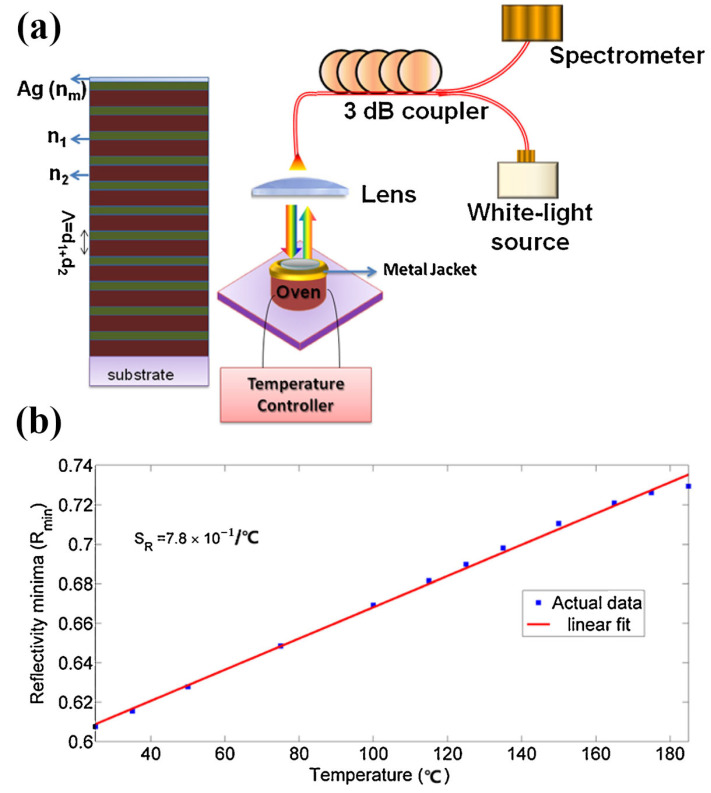
Construction and test result of the plasmonic temperature sensor based on Tamm-plasmon-polariton (TPP). (**a**) Typical metal-distributed-Bragg-reflector structure and the schematic diagram of excitation experiment device. (**b**) Graph of minimum reflectivity versus temperature sensed by temperature-sensor-based Tamm-plasmon-polariton. Reproduced with permission from reference [84]. Copyright 2017 Elsevier B.V.

A type of Tamm-temperature sensor was reported based on surface plasmon resonance (Figure 10), as described by Kumar et al. They proposed a temperature-sensing scheme based on the metal-dielectric structures of the Tamm-plasmon-polariton (TPP). It was used to measure the temperature because of the inclination of the reflection (within the photonic bandgap (PBG) of the distributed-Bragg-reflector (DBR)) caused by excitation of the TPP mode that varied with temperature. The temperature sensitivity of this sensor is 7.8 × 10^−4^/°C in the temperature range of 35–185 °C [84].

A highly sensitive fiber temperature sensor based on photon crystal fibers (PCFs) with periodic microscopic pore structure was reported by Abu Bakar Siddik et al. Compared with conventional fiber sensors, the temperature sensor can adjust structural parameters, such as the thickness of gold, to control sensitivity and performance, which shows high sensitivity at temperatures ranging from 0 °C to 60 °C. The temperature sensor has sufficient temperature monitoring potential in healthcare monitoring and medical treatment [85]. Han et al. have designed a liquid-filled HC-NCF high-sensitivity temperature sensor based on surface plasmon resonance (SPR). Through addition of HC-NCF, this plasmon resonance temperature sensor can significantly improve the temperature sensitivity. By adjusting the structural parameters, such as the thickness of the gold layer, the sensing properties can be effectively improved and the resonant wavelength can be effectively adjusted to the desired band. In the range of 20–40 °C, it has high temperature sensitivity of 2.860 nm/°C [86].

### 3.4. Body Fluid Biosensors

Body fluids, including urine, sweat, saliva, tears, etc., carry abundant proteins and metabolites, which contain the basic information of personal health and the markers of some diseases [87]. Current body fluid biosensors that mostly depend on enzymatic reaction and antigen–antibody interaction are limited by poor stability due to enzymes and antibodies being prone to degrade as time goes on. Plasmonic biosensors can offer special spectral signals for analyte identification without unstable biometric elements [88]. In addition, the SERS signal is not sensitive to strain and temperature changes in the environment, so it does not need to be calibrated in real-time field analysis. Therefore, plasmonic biosensors for detection of body fluids have superiority in rapidity, high stability, low risk of cross infection and large-scale detection, which are of great significance for real-time monitoring of patients’ health [89,90].

**Figure 11:**
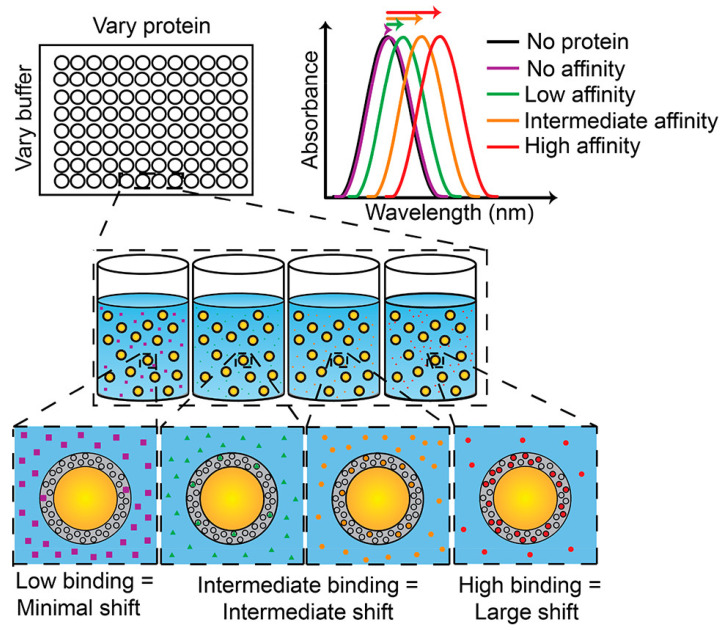
Abridged general view of the AuNS@PNM eye tears biosensor. When the protein concentration or affinity increases, the LSPR wavelength is expected to show a red shift. Reproduced with permission from reference [91]. Copyright 2018 American Chemical Society.

It will increase the refractive index of the plasmonic material around the captured analyte that the plasmonic material decorated with specific molecular recognition agents that can capture the corresponding analyte. Thus, it causes a red shift in the LSPR wavelength and achieves sensing of the analyte. One example is the AuNS@PNM composite material for detecting chronic dry eye in tears proposed by Culver’s group (Figure 11), which shows a large margin of red shift (up to 50 nm) depended on concentration changes in the LSPR wavelength when it is combined with two protein biomarkers (lysozyme and lactoferrin) in human tears. Detection of this plasmonic sensor for tears is convenient and fast: the test can be completed with only a few microliters of tears in a few minutes. It verified the function of this biosensor that the samples from dry eye patients and non-dry-eye patients have been tested successfully, and that the cost of this plasmonic biosensor is low diminishes its threshold of being an affordable tool for rapid screening of chronic dry eye [91].

For sweat detection, a wearable sensor device, which can make real-time and portable sensing come true, is one of the hottest research directions at present [92]. Mogera et al., designed a wearable plasmonic paper microfluidic sensor for continuous and simultaneous quantitative analysis of sweat loss, sweat rate and uric acid (UA) in sweat. The sweat loss and sweat rate can be accurately quantified by the paper microfluidic device, and the concentration of uric acid in sweat can be measured by radiometric SERS intensity, even at concentrations as low as 1 μM. Further, the wearable plasmonic biosensor can attach to the skin directly thanks to its flexibility, stretchability and no toxicity [93].

### 3.5. COVID-19 Biosensors

As a result of the continuous spread of COVID-19 that has brought heavy losses to our physical, mental health and economic development, COVID-19 detection has gradually become normalized in countries around the world [94,95]. It requires rapid and accurate detection to reduce the possibility of community transmission [96,97]. The main strategy for diagnosing COVID-19 is reverse transcriptase polymerase chain reaction (RT-PCR), by which false negative results may be detected due to limitations of the technology itself. Further, plasmonic COVID-19 biosensors have great potential for diagnosis of COVID-19, with the benefits of stability, high sensitivity and abundant functionalization sites and so on [98,99].

**Figure 12:**
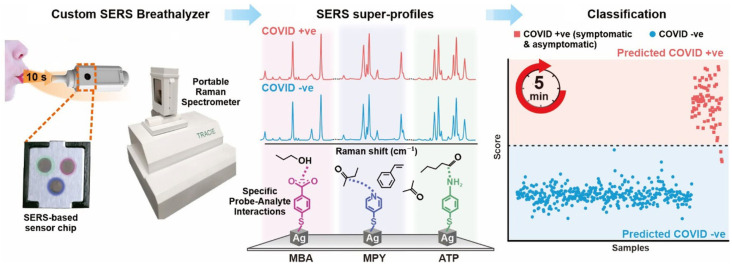
Schematic diagram of using respiratory volatile organic compounds (BVOCs) to identify COVID-19-positive based on plasmonic biosensing. Reproduced with permission from reference [100]. Copyright 2022 American Chemical Society.

Leong and co-workers designed handheld COVID-19 biosensors based on specific spectral variations in surface-enhanced Raman scattering (SERS) due to interactions between respiratory metabolites and multiple molecular receptors on the substrate of silver nanocubes (Figure 12). The COVID-19 biosensor has achieved high sensitivity (96.2%) and high specificity (99.9%) for distinguishing COVID-19-positive individuals within 5 min in detection of 501 participants who need to blow on the sensor for 10 s. What is more, no matter the age, gender, smoking habits or other confounding factors of participants, this biosensor can screen COVID-19-positive individuals fast and effectively. Hence, the COVID-19 biosensor breaks through the limitations of the current traditional gas chromatography–mass spectrometry method for respiration analysis because it can conduct respiration collection and measurement at the same time, making it suitable for large-scale testing in different circumstances [100].

Recently, Qiu et al. reported a two-functional plasmonic biosensor with gold nanoislands (AuNIs) for rapid screening of severe acute respiratory syndrome coronavirus 2 (SARS-CoV-2) on a large scale, combining the plasmonic photothermal (PPT) effect and localized surface plasmon resonance (LSPR) sensing transduction. Using two different incident angles, the plasma resonance of PPT and LSPR can be excited at two different wavelengths. AuNI with complementary DNA receptors based on the self-assembly process of thermal dewetted Au nanofilm was used for detecting specific sequences in SARS CoV-2 through nucleic acid hybridization. The thermoplastic heat generated from PPT can improve the temperature of in situ hybridization, which further assists to promote accurate identification of two similar gene sequences. The plasmonic coronavirus sensor is still sensitive when the virus concentration is as low as 0.22 PM and can accurately capture the desired analyte in the polygenic mixture [101].

## 4. Summary and Outlook

In summary, plasmonic biosensors have various manufacturing methods, such as chemical synthesis of nanoparticles, direct writing lithography, nanosphere lithography, etc., which can be selected and combined according to the advantages and limitations of each method to manufacture more sophisticated sensing units that can be applied to cancer marker detection, gas sensing, temperature sensing, body fluid sensing, COVID-19 detection and other scenarios. Furthermore, plasmonic biosensors can also be extended to more challenging and potential fields such as gene sequencing, which will contribute to risk assessment of complex genotypic diseases and development of personalized medicine. With continuous innovation and development of various micro/nano fabricating methods, as well as wide application of plasmonic biosensors, we are closer and closer to an ideal healthcare approach that can prevent diseases through real-time monitoring of individual body physiological index as well as diagnosing and treating diseases early through convenient screening of disease markers. Nevertheless, materialization of this wonderful prospect requires joint efforts and interdisciplinary support. For instance, the data collected by plasmonic biosensors need to be transmitted quickly to intelligent portable devices to enable real-time analysis of these data [102]. The large datasets generated by plasmonic biosensors also require advanced technologies to process, such as cloud computing, data mining and machine learning [103]. Development and maturity of machine learning can induce plasmonic biosensors to generate advantages and potentialities of real-time monitoring and rapid screening [104]. Well-trained neural network models can create reasoning computational sensing systems and predict the physiological status of users through iteratively analyzing the data-driven sensing results [105]. Furthermore, data security must also be considered in mass data collection. Wearable plasmonic biosensors can be integrated into Internet-of-things applications using blockchain and other data handling protocols, also establishing an ethical regulatory framework for wearable data networks.

## Data Availability

Not applicable.
